# SEQOPTICS: a protein sequence clustering system

**DOI:** 10.1186/1471-2105-7-S4-S10

**Published:** 2006-12-12

**Authors:** Yonghui Chen, Kevin D Reilly, Alan P Sprague, Zhijie Guan

**Affiliations:** 1 Department of Computer and Information Sciences, University of Alabama at Birmingham, Birmingham, AL 35294-1170, USA; 2 San Diego Supercomputer Center, University of California, San Diego, La Jolla, CA 92093-0505, USA

## Abstract

**Background:**

Protein sequence clustering has been widely used as a part of the analysis of protein structure and function. In most cases single linkage or graph-based clustering algorithms have been applied. OPTICS (Ordering Points To Identify the Clustering Structure) is an attractive approach due to its emphasis on visualization of results and support for interactive work, e.g., in choosing parameters. However, OPTICS has not been used, as far as we know, for protein sequence clustering.

**Results:**

In this paper, a system of clustering proteins, SEQOPTICS (SEQuence clustering with OPTICS) is demonstrated. The system is implemented with Smith-Waterman as protein distance measurement and OPTICS at its core to perform protein sequence clustering. SEQOPTICS is tested with four data sets from different data sources. Visualization of the sequence clustering structure is demonstrated as well.

**Conclusion:**

The system was evaluated by comparison with other existing methods. Analysis of the results demonstrates that SEQOPTICS performs better based on some evaluation criteria including Jaccard coefficient, Precision, and Recall. It is a promising protein sequence clustering method with future possible improvement on parallel computing and other protein distance measurements.

## Background

Extracting useful information from biological sequences is an emerging problem with the rapid growth of biological sequences databases. Among biological sequences, protein sequences are an especially interesting category since protein is functionally essential in life and its alphabet is large (20 amino acids). There are several well-known protein databases: Pfam [1] is a collection of protein families and domains which contains multiple protein alignments of these families; National Center for Biotechnology Information (NCBI) [2] protein sequence database is an integrated, text-based search and retrieval system that is very often used in biological research; Swiss-Prot [3] is a protein sequence database which strives to provide a high level of annotation, a high level of integration with other databases, and a minimal level of redundancy; The Protein Information Resource (PIR) [4] serves as an integrated public resource of functional annotation of protein data to support genomic/proteomic research and scientific discovery. These databases are often used as data sources for protein sequence clustering study. In this paper two data sets are from Pfam since Pfam is a semi-automatic protein family database, which aims to be comprehensive as well as accurate and may be used for clustering results evaluation. Swiss-Prot and NCBI protein databases are also applied as data sources because they contain most protein sequences and are very popular in biological research.

As more protein sequences become available, protein structure and function can be better studied with more accuracy and efficiency. Among this research, one of the most important computational methods is sequence clustering [5,6]. The goal of clustering protein sequences is to get a biologically meaningful partitioning. Clustering a large set of protein sequences offers several advantages: Proteins are usually grouped into families based on the sequence similarity clustering, which provides some clues about the general features of that family and evolutionary evidence of proteins; Clustering also helps to infer the biological function of a new sequence by its similarity to some function-known sequences; Moreover, protein clustering can be used to facilitate protein 3-dimensional structure discovery, which is very important for understanding protein's function. Recently developed clustering methods have been successful in clustering a large number of sequences simultaneously. ProClust [7] uses a graph based approach and considers multi-domain sequences; SYSTER [8] overcomes the problem of an asymmetric distance matrix by computing a local pairwise alignment after performing a BLAST [9] search. GeneRage [10] is a fast method for clustering large protein data sets. ProtoMap [11] applies some more elaborate considerations. Among those protein sequence clustering methods, the simplest and most widely used category are based on hierarchical clustering algorithm (single linkage) [12]. It aggregates all the sequences linked by a level of similarity above a given threshold, so that within a cluster any sequence is linked to at least one other sequence. This approach may yield fairly good results, but often a majority of sequences are grouped into one single huge cluster resulting from a massive chain effect due to multi-domain proteins. Blastclust program, one part of BLAST package from NCBI, is an example of single linkage protein sequence clustering . Another category, graph-based clustering algorithms, are also commonly employed due to the clustering quality. BAG [13] is a sequence clustering algorithms based on graph theory and is web available at .

OPTICS (Ordering Points To Identify the Clustering Structure) [14] is a density-based clustering method and is popular because it orders the data into a density-based clustering structure corresponding to a broad range of parameter settings. For density-based methods, it is difficult to decide the input parameters that the algorithm is sensitive to. OPTICS is a good solution to density-based cluster ordering. Although it does not produce clusters explicitly, OPTICS generates an augmented ordering of data sets representing its density-based clustering structure, and this structure can be visualized. Since OPTICS does not limit cluster extraction to global parameters, it is possible to extract cluster information interactively as well as automatically. SEQOPTICS, a sequences clustering system based on OPTICS, is presented in this paper. For any protein sequences clustering method a suitable distance measure needs to be chosen. Some functionally related sequences share little or no discernible sequence similarity and detection of these relationships is difficult. The general practice to carry out protein sequence clustering is based on pair-wise sequence similarity/dissimilarity computed by algorithms such as Smith-Waterman [15]. Some other protein distance measurement such as BLAST [9], FASTA [16] are also very commonly taken in existing systems.

Evaluating clustering results quality is another important issue in clustering analysis. For two-dimensional data, it is clear that one can plot the data and read the distribution to tell how good the clustering results are. But in high dimension data or sequence clustering, direct visualization is normally not feasible. In protein sequence clustering, a popular metric of clustering quality is based on how well the clusters identified by the clustering algorithm match the protein families defined in some database by biological experts [8]. Another method is to compare results of SEQOPTICS with results of some existing methods by using certain validation techniques [17]. Both evaluation techniques are conducted in this paper. In the following the SEQOPTICS clustering system is explained. Then SEQOPTICS is tested with several biological data sets. Visualization results of the clustering are presented. Moreover, the clustering results are analyzed according to the protein families identified by biologist and are also compared with those of two existing methods, blastclust and BAG. Results demonstrate that SEQOPTICS performs better in terms of clustering quality. Some future work needs to be done with the system includes system speed-up and algorithm optimization.

## Methods

**Figure 1:**
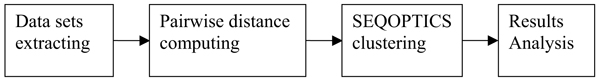
**SEQOPTICS Overview**. This figure depicts four steps in the system: First, data sets are extracted from data sources (mostly protein databases), then mixed and randomized. Three data sources are Pfam, Swiss-Prot and NCBI. Secondly, the pairwise distances between any two proteins are computed. Here a normalized Smith-Waterman score is used. Several other options may be chosen, such as BLAST or FASTA, for distance measure. Thirdly, the OPTICS algorithm is adopted to execute the clustering and the clustering structure is graphically presented. Finally clustering results are analyzed and compared to some other methods based on *Jaccard Coefficient*, *Precision*, and *Recall*.

SEQOPTICS expands the use of OPTICS, a method that has not been used in protein sequence analysis. Figure 1 shows the overview of our method. First, data sets are extracted from data sources (mostly protein databases), then mixed and randomized. Three data sources are Pfam, Swiss-Prot and NCBI. Secondly, the pairwise distances between any two proteins are computed. Here a normalized Smith-Waterman score is used as the pairwise distance. Several other options, such as BLAST or FASTA, may be chosen for distance measure. Then the OPTICS algorithm is adopted to execute the clustering and the clustering structure is graphically presented. Lastly, the clustering results of SEQOPTICS are analyzed and compared to results of some other methods based on some criteria including Jaccard coefficient, Precision, and Recall.

### Data Sets

Four data sets are extracted from different protein repositories as shown in Table 1. Two of them are from Pfam since it is a protein families database and may be assumed as "true" clusters. Pfam multiple alignments come in two forms. In the first form, "seed" alignments are representative, non-redundant sets of sequences that are checked in a manual alignment editor. In the second form, "full" alignments are automatic alignments of every homologous domain [1]. Two other data sets are from NCBI and Swiss-Prot separately. Each protein sequence is labelled by its original notation. This labeling defines the assumed "true" clusters. For example, if a sequence is extracted with "IGA1" from NCBI, then it is labeled as "IGA1" and assumed to be in "IGA1" cluster. The size of each data set ranges from 197 to 319 sequences for testing purpose.

Data set l (see Table 1) contains 197 protein sequences from four different families in Pfam database: 75 sequences of cytochrom_B561 (cytoB), 54 sequences of GABA Receptor (GABAR), 51 sequences of bac_globin, and 17 sequences of glucokinase. Data set 2 contains 268 sequences of three families of globin superfamily from Pfam database: bac_globin containing 51 sequences, IGA1 containing 98 sequences, and band_3_cytochrome (band3) containing 119 sequences. Data set 3 contains 319 sequences from five families in NCBI: 86 cytochrome C (cytoC) sequences, 44 GABAR sequences, 47 GAPDH sequences, 78 GFAT sequences, and 64 GPCR sequences. Data set 4 contains 295 sequences of three families from Swiss-Prot database including: 122 GAPDHs, 62 casein kappas, and 111 globins. For each data set, protein sequences from different families are mixed and randomized to minimize the effect of pre-defined manual clustering.

### Computing distance

Our approach, consonant with others, starts with a distance measure. When data sets are from different protein families, it is a common practice to use a normalized pairwise local alignment score by Smith-Waterman dynamic programming algorithm. There are several parameters in Smith-Waterman, for example, scoring matrix, open gap penalty and extending gap penalty. Various scoring matrices including BLOSUM50 and PAM250 have been tried. BLOSUM50, which is also used in FASTA [16], is used as default in this paper. The default open gap penalty taken is 12 and the extending gap penalty is 2. The similarity score between two protein sequences is then calculated by the following normalization formula:



where *S*(*a*, *b*) is the Smith-Waterman local alignment score between two sequences *a *and *b*; *S*(*a*, *a*) is the similarity score of sequence *a *to itself; *S*(*b*, *b*) is the score of sequence *b *to itself; and *SN*(*a*, *b*) is the normalized score.

The distance between two protein sequences is defined as:

*Distance*(*a*, *b*) = 1 - *SN*(*a*, *b*);

With this normalization, every distance score is between 0 and 1. If other scoring methods are used instead of Smith-Waterman, the distance measure needs to be adjusted appropriately.

### OPTICS clustering

Some preliminary remarks on OPTICS have been given in the introduction. Some definitions of the concepts used in OPTICS are described as follows: An object *p *is in the *ε-neighborhood *of *q *if the distance from *p *to *q *is less than *ε*; A *core object *has at least *MinPts *neighbors in its *ε*-neighborhood. The *reachability distance *of *p *is the smallest distance such that *p *is density-reachable from a core object *o*. A cluster is a set of density-connected objects which is maximal with respect to density-reachability. A *reachability plot *is a bar chart that shows each object's reachability distance in the order the object was processed which demonstrates the cluster structure of data. The final clusters can be extracted by either *ε*-cutoff or steepness of the plot. For more detailed information about OPTICS algorithm, please refer the original paper [14].

SEQOPTICS is implemented with a distance measure of sequences based on Smith-Waterman algorithm. The core OPTICS part was tested with the data sets from OPTICS authors. Two parameters need to be chosen, *ε *and *MinPts*. In this paper, since the distance between any two protein sequences is between 0 and 1, a single *ε *for all data set may be used, for example, *ε *is set as 0.99, which is slightly smaller than 1. The *MinPts *used here is 10 based on the measurement of the experimental data sets. For the whole protein database, *ε *can still use any value between 0.95 to 0.99, *MinPts *should be set as the average number of sequences in a family.

There are two main advantages to apply OPTICS in protein sequences clustering analysis: 1) OPTICS can find the local density region; 2) OPTICS produces an augmented ordering of the sequences representing its density based clustering structure and this ordering can be visualized, for example, in the reachability plot. The cluster ordering actually contains information about every cluster, i.e., OPTICS enables the extraction of not only "traditional" cluster information, but also intrinsic clustering structure.

## Results and Discussion

SEQOPTICS is applied to cluster the experimental data sets. Visualization results are presented also. These provide some clues about clustering structure. The final density-based cluster sets are defined from the ordering reachability distance. To evaluate the resulting clustering set's biological accuracy, we need to compare it to a "true" cluster set. However, there is no generally accepted "true" cluster set. All automatical protein clustering methods are based on "all against all" sequence comparison and real clusters need to be verified by biological expertise. Since it is impossible to have "real" clustering, the original database clusters are assumed to be "real" clusters. That is the way that most automatic protein clustering does. For example, all sequences from the *glucokinase *family of Pfam are considered in the same cluster.

### Visualization of the cluster structure

**Figure 2:**
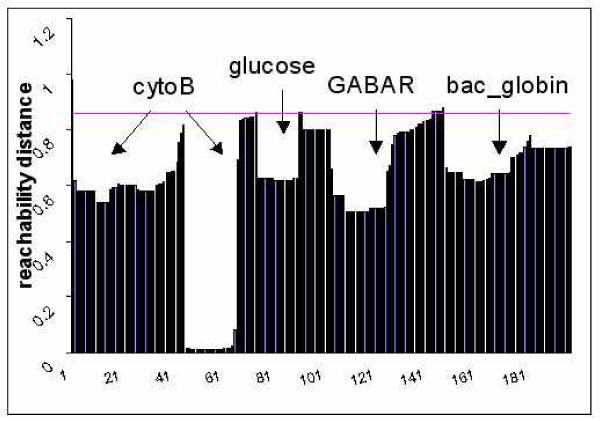
**Cluster structure of data set 1 (Pfam)**. Valleys represent clusters and the purple line stands for the cutoff value for extracting clusters. This figure shows 5 valleys corresponding to 4 protein families.

**Figure 3:**
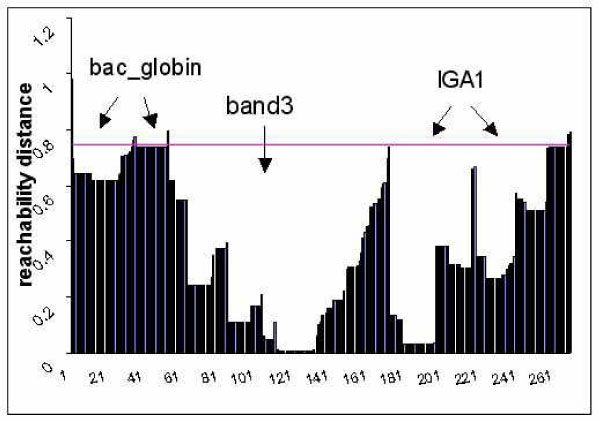
**Cluster structure of data set 2 (Pfam)**. Valleys represent clusters and the purple line stands for the cutoff value for extracting clusters. This figure shows 4 valleys corresponding to 3 protein families.

**Figure 4:**
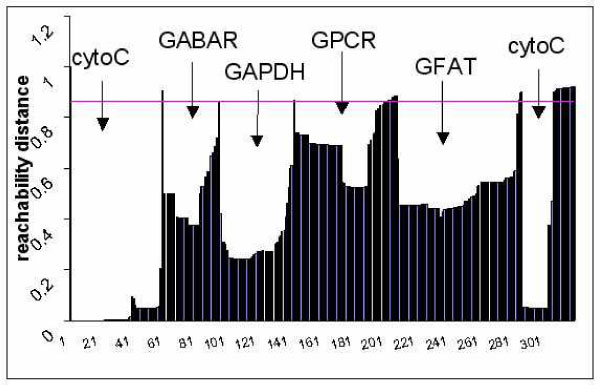
**Cluster structure of data set 3 (NCBI)**. Valleys represent clusters and the purple line stands for the cutoff value for extracting clusters. This figure shows 6 valleys corresponding to 6 protein families.

A reachability distance plot is made for each data set. These plots are shown in Figure 2, 3, 4, and 5. In each figure, the horizontal axis represents the ordering of each sequence, the vertical axis represents the reachability distance, and each valley stands for a cluster set.

**Figure 5:**
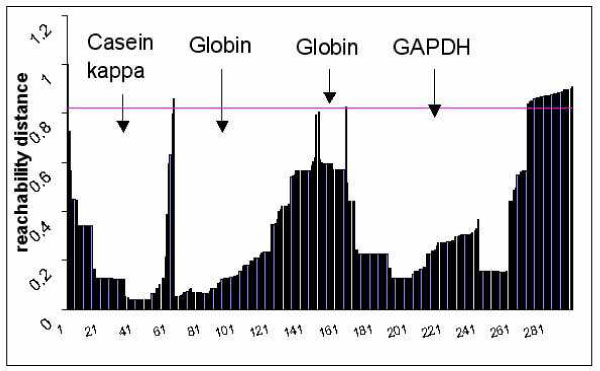
**Cluster structure of data set 4 (Swiss-Prot)**. Valleys represent clusters and the purple line stands for the cutoff value for extracting clusters. This figure shows 4 valleys corresponding to 3 protein families.

For data set 1, there are five valleys in Figure 2: The first two valleys are composed of sequences from cytochrom_B562; The third valley consists of sequences from glucokinase; The fourth valley contains sequences from GABAR family; The fifth valley are sequences from bac_globin family. For data set 2, there are three valleys in Figure 3: The first one is composed of sequences from bac_globin; The second valley is composed of sequences from band3 family; The third valley contains only sequences from IGA1. For data set 3, there are six valleys in Figure 4: The first one and last one contain only cytoC sequences; The second valley contains only sequences from GABAR; The third valley contains sequences GAPDH; The fourth valley contains GPCR sequences; The fifth valley contains only GFAT. For data set 4, there are four main valleys in Figure 5: The first valley contains only casein kappa sequences; The second and third valley contain exclusively globins; the fourth valley is composed of GAPDHs.

Those figures shows that each valley contains exclusively one sequences family. Assume a new protein sequences is found: you may throw it into a pool of sequences and find which valley it is located in, then it is very possible that this new protein is from the same family as those in the same valley.

### Extraction of the clusters

The final density-based clusters are extracted by using a cutoff value. For example, in Figure 2, the cutoff value is set as 0.860 (shown as the line *reachability distance *= 0.860). Under this cutoff condition, each valley between two sequences with *reachability distance *higher than the cutoff is a cluster. The sequence starting a valley with *reachability distance *higher than the cutoff is also in the same cluster as rest sequences in the valley. Any sequence with *reachability distance *higher than the cutoff is noise if it does not start a valley. Therefore, in Figure 2, there are four clusters give the cutoff value 0.860, which is decided by experience. Similarly, there are four clusters in Figure 3 given cutoff 0.745, six clusters in Figure 4 given cutoff 0.860, three clusters in Figure 5 given cutoff 0.820.

### Validation of the cluster set

To evaluate the resulting cluster sets with respect to its biological accuracy, the following problems need to be addressed:

• There is no generally accepted "true" cluster set. That is to say, those "true" clusters are always "biased". However, if appropriate data source is chosen, then the "bias" can be limited.

• There are some automatically generated cluster sets and some manually generated cluster sets. Those cluster sets are usually organized in "families", thus make the validation easier.

Automatically generated cluster sets are not necessarily biologically correct. They are normally based on all-against-all sequence comparisons. Pfam is an example of this category. Pfam is a large collection of common protein domains and families based on the UniProtKB/Swiss-Prot Protein Knowledge base. Pfam seeds contain the seed alignments of the families and therefore are more accurate than general Pfam families. In this paper Pfam seeds are used for testing to reduce "bias" of "true" cluster.

NCBI is probably the most complete protein sequences database. UniProtKB/Swiss-Prot provides a high level of annotation (such as the description of the function of a protein). SEQOPTICS data are extracted from NCBI and SwissProt since they are probably two most complete databases in biological research. Those extracted data are further manually pre-processed, i.e., those protein similar in annotation and sequences are selected so that "bias" is reduced.

As has been mentioned earlier in this paper, the original database clusters are considered as the "true" clusters against which the algorithm derived clusters are evaluated. Based on this assumption, several statistics metrics are used to evaluate the result.

According to Krause's PhD thesis [8], a cluster set of *n *data points from the experiment can be represented by the  values in a triangular matrix *M*, where for *i *<*j*, *M*_*ij *_= 1, if and only if *i *and *j *are in the same cluster and *M*_*ij *_= 0 otherwise. If *T *is a matrix of "true" clusters, the two cluster sets ("true" and "experimental") can be compared based on the following numbers:

• *a *is "true positive," i.e., the number of sequence pairs clustered together in both sets, which can be denned as: *a *= |(*i*, *j*)|*M*_*ij *_= 1 ∧ *T*_*ij *_= 1, *i *<*j*|

• *b *is "false negative," i.e., the number of sequence pairs clustered together in the true cluster set, but not in the clustering solution, defined as: *b *= |(*i*, *j*)|*M*_*ij *_= 0 ∧ *T*_*ij *_= 1, *i *<*j*|

• *c *is "false positive," i.e., the number of sequence pairs clustered in the current solution, but not in the true cluster set, defined as: *c *= |(*i*, *j*)|*M*_*ij *_= 1 ∧ *T*_*ij *_= 0, *i *<*j*|

• *d *is "true negative," i.e., the number of sequence pairs not clustered in either current solution or the true cluster set, defined as: *d *= |(*i*, *j*)|*M*_*ij *_= 0 ∧ *T*_*ij *_= 0, *i *<*j*|

There are many validation techniques as cited in reference [17]. In this paper three parameters are applied based on the above definitions: *Precision*, *Recall *[18,19], and *Jaccard Coefficient *[20].

**Figure 6:**
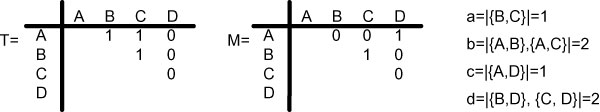
**Comparison of Two Cluster Sets *T *and *M***. By counting those sequence pairs clustered in the same way and those clustered differently in *T *and *M*, the *Jaccard Coefficient *is: *S*(*T*, *M*) =  = 0.25. the *Precision *is: *P*(*T*, *M*) =  = 0.5, the *Recall *is: *S*(*T*, *M*) =  = 0.33.

*Precision *is defined as:



*Recall *is defined as:



*Jaccard Coefficient *is defined as:



All three parameter values range between 0 and 1. The better the clustering, the bigger the values. In a perfect clustering which is identical to the true cluster, *P *= 1, *R *= 1, and *S *= 1. Most existing sequence clustering methods perform well in terms of *Precision *but not in *Recall*. Figure 6 shows an example of calculating three parameters. These three parameters are also calculated based on our experimental results as shown in Table 2.

Same data sets are tested with two other clustering methods, BLASTClust [9] and BAG [13], using default parameters of these methods. BAG is a graph based clustering method and graph clustering is used in some popular protein clustering methods including ProClust [7], SYSTERS [21]. BLASTClust is chosen because it is from NCBI BLAST package and is a widely used hierarchical sequence clustering method. The validation of our experiments is based on *Jaccard Coefficient*, *Precision *and *Recall *comparison values as shown in Table 2. Table 2 demonstrates that SEQOPTICS produces good results relative to each original cluster set in terms of *Jaccard Coefficient*. Every SEQOPTICS *Jaccard Coefficient *is higher than 0.65 and the highest being 0.85. It is also seen in the table that SEQOPTICS outperforms BAG and BLASTClust on all the data sets chosen on this criterion. The performance with BAG exceeds BLASTClust for the same reason. However, BAG and BLASTClust tend to give more clusters than the "true" clusters, explaining why the *Precision *of those two methods on all data sets are 1. Take Pfam1 as an example, SEQOPTICS gives 4 clusters, BAG results in 24 clusters and BLASTClust gives 121 clusters. Therefore, BAG and BLASTClust give high *Precision *values and low *Recall *value. But neither of these two performs well in terms of *Recall*. Overall, SEQOPTICS performs better than BAG and BLASTClust and seems a promising method in terms of both clustering quality coupled with its graphical representation of clustering structure.

Although manual cluster sets combined with biological experiment and the experts' information are the ultimate validation criterion, computer-evaluation can be considered a tool at the disposal of experts in evaluating clustering results.

## Conclusion

In this paper we described a prototyped system, SEQOPTICS, for protein sequences clustering as shown in Figure 1. A core portion(phase) of the system is based on OPTICS clustering and visualization method, which we believe is being used here for the first time for protein sequence clustering. Prior to this phase, it is necessary to compute the distance between (protein) sequences. A normalized Smith-Waterman score is used in this paper to compute the required distance. The last system phase, Results Analysis, demonstrates adequacy of our approach for small-scale data and the usefulness of the cluster structure visualization. According to Ankerst [14], one good feature of OPTICS is that it does not limit oneself to a single set of global parameters. An augmented cluster ordering contains information equivalent to density based clusterings corresponding to a broad range of parameter settings; as such, the cluster ordering is a versatile base for both automatic and interactive cluster analysis. A second good feature lies in the visualization of the data set distribution. Depending on data set size, one can either represent the cluster-ordering graphically for small data sets, or, employ an alternate technique (appropriate) for large data sets. This paper demonstrates that in SEQOPTICS the visualization of cluster structure is meaningful. The time complexity of Smith-Waterman is O(*n*^2^*l*^2^), where *n *is the number of sequences and *l *is the average length of the sequence. The time complexity of OPTICS is O(*n*^2^) in the implementation. Therefore the total time complexity is O(*n*^2^*l*^2^). This is an expensive method if Smith-Waterman is the only choice of the distance measure. Fortunately there are some other options for the distance between two protein sequences, such as BLAST or FASTA which will dramatically decrease the overall time complexity. SEQOPTICS has proved its value for handling small data sets (<1000 sequences)  in this paper. If this system is applied to a large data set, such as a whole database, future improvements are necessary to make it more successful. The following directions are considered in future: 1) use some other distance measure for protein sequence distance, e.g., BLAST or FASTA; 2) apply parallel computing tools, for example, Message Passing Interface(MPI) for large data sets; 3) implement visualization techniques for large data sets; 4) consider incremental cluster ordering algorithms since protein databases are very frequently being updated.

## Authors' contributions

We created a system (SEQOPTICS) which applies an existing clustering method into protein sequence clustering and evaluated the clustering results.

## Figures and Tables

**Table 1:** Protein Sequences Data Sets

Data set	1	2	3	4
From	Pfam (197)	Pfam (268)	NCBI (319)	Swiss-Prot (295)

Families	cytoB(75)	bac_globin(51)	cytoC(86)	GAPDH(122)
	GABAR(54)	IGA1(98)	GABAR(44)	casein kappa(62)
	bac_globin(51)	band3(119)	GAPDH(47)	globin (111)
	glucokinase(17)		GFAT(78)	
			GPCR(64)	

Note: The number in parenthesis is the number of sequences in each family

**Table 2:** Comparison of Clustering Results

Data Set	*BLASTClust*			*BAG*			*SEQOPTICS*		
	*S*	*P*	*R*	*S*	*P*	*R*	*S*	*P*	*R*

l(Pfam)	0.05	1.00	0.05	0.27	1.00	0.27	0.83	0.99	0.84
2(Pfam)	0.04	1.00	0.04	0.20	1.00	0.20	0.85	0.98	0.87
3(NCBI)	0.11	1.00	0.11	0.60	1.00	0.60	0.66	0.82	0.78
4(Swiss-Prot)	0.06	1.00	0.06	0.50	1.00	0.50	0.81	0.99	0.82

Clustering results of four data sets by three methods according to three parameters: *S(Jaccard Coefficient)*, *P(Precision)*, *R(Recall)*. It shows that SEQOPTICS outperforms BAG and BLASTClust which tend to give more clusters than "true" clusters.
